# Supplementary material for: Social Responsiveness and Psychosocial Functioning in Adults with Prader–Willi Syndrome
Source: J Clin Med. 2022 Mar 5;11(5):1433. doi: 10.3390/jcm11051433 (PMC8911114; doi:10.3390/jcm11051433)
Supplement: Supplementary file 1 [file jcm-11-01433-s001.zip › jcm-1588696-supplementary.pdf]

Journal of
Clinical Medicine

MDPI

Supplementary Materials

Degrees of difficulty in the Personal and Social Performance (PSP) scale (Morosini et al., 2000; García-Portilla et al., 2011), with adapted scores.

Patients' degree of difficulty in each main area was classified in one of six categories: absent; mild; manifest, but not marked; marked; severe; or very severe. One set of operational criteria were used to judge the degree of difficulty for the first three main areas (*Socially useful activities*, *Personal and social relationships*, and *Self-care*), and a different set of criteria were used to judge the fourth main area (*Disturbing and aggressive behavior* area) (Morosini et al., 2000).

As described by Morosini et al. (2000) and García-Portilla et al. (2011), we used the following criteria used to classify the degree of difficulty in the first three main areas (see below). For the purpose of our study, we assigned the following scores to the descriptions used in the above-mentioned studies:

(0) absent,

(1) *mild*, operationalized as unmanifested difficulties that could be identified only by someone familiar with or close to the person.

(2) *manifest, but not marked*, operationalized as difficulties that anyone could clearly identify but that do not interfere substantially with the person's ability to perform his/her role in that area, given the person's sociocultural context, age, sex, and educational level.

(3) *marked*, operationalized as difficulties interfering heavily with role performance in that area. Importantly, however, "the person is still able to do something without professional or social help, although inadequately and/or occasionally; if helped by someone, he/she may be able to reach the previous level of functioning". "Occasionally" is defined as "occurring three or more times in the reference period or occurring even less than three times but in circumstances and/or with such a previous history to convince the rater that there is a risk of recurrence within the next 6 months".

(4) *severe*, operationalized as difficulties that render the person unable to perform any role in the main area being evaluated without professional help or that lead the person to a destructive role; however, this degree of severity does not pose a risk for the person's survival.

(5) *very severe*, operationalized as impairments and difficulties that endanger the person's survival.

As described by Morosini et al. (2000) and García-Portilla et al. (2011), we used the following criteria used to classify the degree of difficulty in the fourth main area (*Disturbing and aggressive behavior*). For the purpose of our study, we assigned the following scores to the descriptions used in the above-mentioned studies:

(0) absent,

(1) *mild*, corresponding to mild rudeness, unsociability, or whining.

(2) *manifest, but not marked*, corresponding to behaviors such as speaking too loudly or in an overly familiar manner, or eating in a socially unacceptable manner.

(3) *marked*, corresponding to behaviors such as insulting others in public, breaking or wrecking objects, acting frequently in a socially inappropriate but not dangerous way (e.g., stripping or urinating in public).

(4) *severe*, corresponding to behaviors such as frequent verbal threats or frequent physical assaults, without the intention to inflict severe injuries or possibility of inflicting such injuries unintentionally.

(5) *very severe*, corresponding to frequent aggressive acts, aimed at causing or likely to cause severe injuries.

Morosini et al. (2000) also described some specific concerns about the interpretation of suicidal acts or risk, but we did not consider these aspects in our study.

Higher scores in total PSP represent better global personal and social functioning. These levels could be classified in four groups:

- Scores between 91 and 100 indicate excellent functioning in all four main areas.
- Scores between 71 and 90 indicate mild difficulties in functioning.
- Scores between 31 and 70 indicate manifest to marked difficulty in functioning.
- Scores ≤30 indicate severe difficulty in functioning that requires intensive supervision.

Description of the medical and psychopharmacological treatments:

- Among the most common medical treatments administered were oral antidiabetics (n=6), insulin (n=3), antihypertensives (n=4), lipid-lowering agents (n=2), levothyroxine (n=3), and other hormonal treatments (n=4) in different combinations.

- Among the most common psychopharmacological treatments administered were different antipsychotic medications (n=7; median chlorpromazine equivalent 100 mg/d, range 25–280 mg/d), antidepressants (n=14; fluoxetine in 4, sertraline in 4, other antidepressants in 6), benzodiazepines (n=3, median diazepam equivalents 2.5 mg/d, range 2.5–5 mg/d), topiramate (n=10, median dosage 250 mg/d, range 50–500 mg/d), and zonisamide (n=3, median dosage 50 mg/d, range 50–350 mg/d).
